# A Criticism of Some Recent Utterances on Ectopic Gestation

**Published:** 1890-01

**Authors:** Lawson Tait

**Affiliations:** Professor of Gynecology, Queen’s College, Birmingham, Eng.; 7 The Crescent


Buffalo Medical and Surgical Journal

VOL. XXIX. JANUARY, 1890. No. 6.

### Original Communications.

### A CRITICISM OF SOME RECENT UTTERANCES ON ECTOPIC GESTATION.

BY LAWSON TAIT, F. R. C. S. E.,

Professor of Gynecology, Queen's College, Birmingham, Eng.

IT IS now nearly twelve months since I laid before the profession my completed views on this interesting subject, and I now take an opportunity of noting some of the criticisms of the work which occupied much of my time and thought for fifteen years before I published my completed conclusions.

In the first place, I have to acknowledge, with gratitude, the almost uniform acceptance of my views by American and Continental authorities; indeed, in the case of German writers, the acceptance is so complete that their British originals have been entirely absorbed into a Teutonic solidarity. American writers, on the contrary, and as is the custom in their country, have fully rendered credit where they believed credit was due, and I have nothing to give them back but the fullest acknowledgment of their generosity. Of English writers, I have, with two exceptions, to speak with as fully grateful recognition. A few criticisms have been offered of a perfectly fair kind, upon points where there is either, as yet, room for difference of opinion, or upon others where, by reason that I did not see the necessity for fuller exhibition of evidence, I did not as completely state my case as I should have done. My excuse is, that the field was a wide one and a new one; and that in it I made roads not only fresh, but leading, almost without exception, in directions wholly at variance with those previously accepted, alike in the pathology of ectopic gestation and its treatment.

The writers to whom I wish to invite attention, as exceptions most remarkable, occupy columns of the *Lancet*,—an organ of professional opinion whose good word it has never been my lot to merit, for reasons, I suppose, I shall never understand.

In its issues for the 12th and 19th of October, 1889, that journal deals with the subject of "Ectopic Gestation," in two "editorials" under the title of "Extra-uterine Gestation," and begins its discussion by a frank admission "that many alleged cases were not extra-uterine, but which were due to pregnancies occurring in a malformed uterus." The anonymous writer has not yet mastered the most indisputable conclusion of all my writing, that we ought at once to give up the term "Extra-uterine," for the very reason he gives, coupled with the other of which he is not ignorant, that the most deadly form of ectopic gestation is truly *intra-uterine*.

This is hardly the place to discuss that much-vexed question of journalism, whether articles ought to be signed or not. Most leading articles in the columns of the *Lancet*, and of other medical journals, deal with subjects more or less of a political nature; and even in medical politics there is sufficient nonsense written to make it advisable that the names of the authors should be withheld. But, as the recognized editor of the *Lancet* is not a gentleman in whose life the study of ectopic gestation can possibly have been a leading feature, it needs but scant gynecological wisdom to see that an unsigned article on such a subject in that journal, can have but little weight. The writer has such curious ideas of dealing with his subject fairly, that he makes one big jump from the confused jumble of the classification of Dezeimeris to the article of Hart and Carter; and he is so deficient in patriotism that he never even once brings in the name of the writer whose book has evidently been the cause of the articles, and whose views he has not even taken the trouble to understand. He, evidently, has taken some second-hand misrepresentation of them from the German, for he says that Kussmaul proved that "many" alleged cases were not extra-uterine, but were due to pregnancies occurring in a malformed uterus; whereas the fact is, that only two cases, in the whole literature of the subject, answer the conditions of this anonymous writer, against hundreds, if not thousands, to which the allegation does not apply.

The policy of the *Lancet* has been to ignore the existence of the British Gynecological Society, and the last editor personally assured me that this was a policy that the journal in question would continue to follow. This mistake is proving a misfortune for its writers as well as for its readers.

As a further illustration of the blundering into which such ostrich-like blindness must inevitably lead, let me quote another sentence,—another blunder involved in the use of the term "extra-uterine." "It may be briefly said," continues my critic, "that the differential diagnosis (between extra-uterine and cornual pregnancy, that is—the pregnancy occurring in a malformed uterus,) depends on the point of origin of the round ligament of the uterus; since this springs from the uterus, and therefore is internal to the tube, a gestation external to its origin must be tubal, internal to its origin, uterine." Now, our interstitial pregnancies (a more common form, and far more fatal than cornual pregnancy in a bifid uterus,) are both tubal and uterine; they are not extra-uterine, but most certainly *are* ectopic; so that we must conclude that our anonymous author has not understood the prime steps of the syllogism. He has read my quotation from Kussmaul and Sir William Turner, without reading (or, at least, without being able to understand,) how beautifully they fit in as bricks in my edifice; or, he has, as I said before, taken some second-hand German appropriation, instead of the original work.

This conclusion is inevitable, when a little further on he assumes that intra-peritoneal hematocele is known to occur as a thing of itself, and arising from any other cause than traumatic hemorrhage from an injured organ, such as kidney, liver, or distended broad ligament, rupture, or aneurism, or rupture of a tubal pregnancy. If it does, where are the cases? Who are the authorities?

Upon some of the questions of treatment the anonymous writer, under shelter of the editorial "we," gives expression to some opinions which are the chief reason of my referring to his article at all. "In my opinion," he says, (concerning the treatment of the case in the later months of pregnancy,) "in the face of the tremendous maternal risks, it is bad sentiment, bad morality, and bad surgery, to regard the life of the fetus for a moment." We may leave sentiment out of the question, whether good or bad, but when we come to discuss a question as one of morals, we must, at least, listen to the voice of mankind. In Europe and America we are governed by the code of morals appertaining professedly to the Christian religion. The morals of this religion, as well as its theology—though not so much—vary according to sectarian view. The oldest body, by far the largest and most influential, the only body which has deliberately discussed this question—I mean the Roman Church—has decided that the life of the fetus *must* be considered; and no other sect has formally, or even casually, discussed this question. The morals of the *Lancet* are therefore bad, I may say very bad, upon this point.

When an anonymous writer says it is bad surgery to discuss the question of the life of the fetus, I claim the right to know what authority speaks! Has he ever operated in such cases? Does he know anything about it at all? I can speak and have spoken, and the authority of the experience with which my utterances are made is the most extensive that has yet been offered, and my conclusion is that it is safer to the mother to consider the life of the child; and no surgeon will say that the glory to his art is not greater, in any operation, to save two lives rather than one.

Even an anonymous writer in the *Lancet* might take the trouble to test the accuracy of such a statement as that "It has been proposed to cut off the cord short and close the wound; but this has not gone beyond the stage of proposal." This has been done, and would have been successful had not the operator trusted to the delusive visions of Lister. That the editor of the *Lancet* may, with advantage to his readers, make some change in his obstetric staff, is evidenced by the final and ludicrous blunder contained in the statement that "The position of the placenta in these cases may sometimes be determined by palpation, but not by the (incorrectly named) 'placental souffle,' which has nothing whatever to do with the placenta, and in all carefully observed cases has been found to be absent over its site." In the one case in my experience, where it was possible to make the observation, the only case where the existence and position of the placenta could be determined at all, it was carefully mapped out by the souffle, and it was found at the operation that my mapping out was perfectly correct.

The number of the *Lancet* in which this remarkable article appears, opens with Mr. Bland Sutton's able and pointed essay on "Intellectual Blindness," and our anonymous writer concludes by saying that "The discussion of the subject has been somewhat burning in many quarters. It is, above all things, desirable that personalities should be strictly avoided." Dr. Matthews Duncan therefore begins his own article in the *Lancet* with the remarkable sentence, that "it is only audacious ignorance that could give clear and decided teaching on the theory and practice of extra-uterine gestation;"—whatever that may mean, whatsoever the *practice* of extra-uterine gestation may be. "And," he continues, "it is clearness and decision, when they are fairly attainable, that should characterize the clinical teaching of youth."—leading to an assumption that, when he dare indulge, the great obstetric physician of St. Bartholomew's is not free from audacious ignorance.

Dr. Duncan tells us "that the theory and anatomy of it (extra-uterine gestation—I fear he will never be led to adopt the scientific nomenclature, seeing we owe it to Dr. Barnes) have made some progress, due chiefly to improved anatomical methods, especially homalographic frozen sections, and to the many laparatomies which are now performed in this disease." Dr. Duncan must know, for I happen to be certain that he has read my book, that every fact but one of the anatomy, and abundant confirmation of my theory of ectopic pregnancy, was obtained from my abdominal sections years before the fortunate sections, obtained by Hart and Carter, established the proof in such a way that even Dr. Duncan could no longer ignore the facts. The one fact in exception was the finger-glove extension of the peritoneum retained from the fundus of the uterus. "But," says Dr. Duncan, "laparatomies do, in most cases, give imperfect information as to anatomy. Often, indeed, they mislead." Perhaps that is so in Dr. Duncan's experience; indeed, I am sure it is so, but I submit it depends upon who performs the operation. Before operations were performed by the younger generation of gynecologists for diseases of the Fallopian tubes, we listened in patient confusion to Dr. Duncan's prelictions on *parametritis* and *perimetritis*. Now, we give him no heed, for abdominal section has brushed aside all the nonsense he taught. In similar confusion, a striking contrast to the clearness which justifies the efforts of audacious ignorance, Dr. Duncan goes on to jumble up, for the benefit of the ingenuous youth he is paid to mislead, the ancient and modern classification of ectopic gestation, and tells us that "there are two sets of kinds of extra-uterine" gestation with which another which is not extra-uterine must be classed,—our old friend the cornual pregnancy,—continuing, "Of this you have here a museum specimen." I wonder where he got it, for there is none in the Museum at St. Bartholomew's, and only one in London—not familiar, I am sure, to Dr. Matthews Duncan, for it comes from a hated laparatomy. "This (cornual) pregnancy . . . . is generally easily made out on dissection post-mortem" (!!!) Of the three museum specimens known to me, one was completely misunderstood when examined first, and so were the other two, being only unraveled by the enthusiasm of a young demonstrator of anatomy, thirsting for the fame which has since deservedly came to him—Sir William Turner. Of these two cases Dr. Duncan knew nothing till they were republished in my book, and of the third I am sure he knows nothing now, for in all his voluminous writings I cannot find a word about this cornual pregnancy about which he speaks with such flippant familiarity, until I come to this unhappy lecture in the *Lancet*. Besides there being the "two sets of kinds" of extra-uterine pregnancy so clearly defined by Dr. Duncan, he tells us that there are secondary variations: "Thus, an ovarian pregnancy may become ovario-tubal. An interstitial pregnancy may become tubo-uterine" and so a cornual pregnancy behaves like an extra-uterine. "Again, a tubal pregnancy may become extra-peritoneal, the tube opening up the broad ligament, probably generally by rupture where the folds of the ligament separate to enclose the tube. You know that an ovarian tumor may in like manner open up the broad ligament and become extensively extra-peritoneal.'' Turning to Dr. Matthews Duncan's Clinical Lectures, this extraordinary mass of nonsense is explained. Dr. Duncan does not yet know the difference between a cystic tumor of the ovary and an embedded cyst of the broad ligament, and I feel hopeless that he ever will. And, to cover all this mass of confusion, the sacred name of Spiegelberg is invoked, chiefly for the purpose of publishing the fact that his book was mistranslated by one of Dr. Duncan's pupils.

Some of Dr. Duncan's assertions on the subject of ectopic gestation are made with all the clearness and decision which he claims for audacious ignorance, and my mind is greatly puzzled whether the greatest respect is due to audacious ignorance or ignorant audacity. "The original site," says Dr. Duncan, of an extra-uterine gestation, "is determined by the insertion of the placenta. There is no other site of placental insertion but the original site—no reason to believe that the placenta ever does or ever can change its site. It cannot be transplanted. Authors of eminence have believed that it can be transplanted,'' etc., but these poor persons, of whom I am one, are waved behind him, by a Podsnappian gesture, and the poor placenta must, perforce, return to its original site. But I have caught it in the act, over and over again, surreptitiously leaving its original site and fastening its octopus-like villi into strange and new places. I have caught it before it had time to retrace its steps and hide Dr. Duncan's shame. "*E pursi muove.*" Dr. Duncan continues, "Were transplantation of placenta possible, many cases of abdominal pregnancy, or all cases, might be called secondary; and this would increase the predominance of tubal, and give a fine appearance of simplicity." This is precisely the case, and it is the tremendous simplicity of the theory, which is at once its recommendation and its gain. It is the same simplicity which floors Podsnap, who comprehends neither simplicity of motive nor directness of action.

"These remarks," continues our lecturer, "are to be applied to extra-peritoneal pregnancy also—that is, when the ovum grows between the layers of the broad ligament, separating them and reaching the parametric cellular tissue. The placenta cannot be transplanted into the cellular tissue.'' These two sentences, of course, contradict one another, and Podsnapery defeats itself. It has seen Hart and Carter's plates without understanding them,—it is intellectually blind.

A few more of Dr. Duncan's views may be commented on, because they are mere statements, in direct opposition to every known fact. Thus, " A tubal gestation generally ruptures, but not always." Can he produce a specimen, after the period of rupture (say the fourteenth week), which has not ruptured? Dr. Duncan has published one case of extra-uterine pregnancy of a character quite unique, but its value does not lie in this direction, as I have had occasion to show. Towards the conclusion of his lecture faint gleams of light seem to have permeated his mind, but, even then, so obscured by prejudice that they must have misled rather than helped his hearers. " In general, it is laparatomy" (by which he ought to have explained to the university graduates, among his audience, that he meant linear abdominal section, and not an incision in the neighborhood of the kidney,) " you have to consider. When the fetus is already viable or further advanced, you will think of early laparatomy, and keep in mind the great danger of hemorrhage in the separation of the placenta, which you have no satisfactory means of arresting." Dr. Duncan is not an authority on abdominal surgery, and, therefore, he ought to accept the statements of those who are, that there exists perfectly satisfactory means of arresting such hemorrhage in the use of perchloride of iron.

His conclusion is still more remarkable, when he formulates his opinion as to " What will be the ultimate result of the present enthusiasm for abdominal surgery, no one can foresee. In extra-uterine gestation much has yet to be done before the utility of laparatomy can be well defined. We need more knowledge of the natural progress of the disease, more knowledge of the anatomy, more experiments in laparatomy. Already much is surely to be gained by judicious laparatomy, and more is to be expected in the future."

About twenty years ago, a decayed old ostler of one of the old city inns used to hang about the scenes of his youth. Broken-down stages lay about the yard, useless, and the old man went about apologizing to such stray visitors as, knowing him not, would listen to him, apologizing for some strange and unforeseen accident which delayed the arrival of the York coach. Poor old Judkins!

How strangely different the words of the modern railway conductor, Lusk by name. His recent paper is one long and judicial examination, with final exordium, not of what I claim as my own, but what I claim for British surgery, the merits of which are so grudgingly withheld, and the views of which are so stupidly misrepresented by Dr. Matthews Duncan.

Within the last few days, we have had what may be termed, by a stretch of courtesy, a debate on Ruptured Tubal Pregnancy at the

Royal Medical and Chirurgical Society, based on a short paper contributed by Mr. Bland Sutton, and upon this paper and some of the speakers in the debate, I wish to make some comments.

In the first place, Mr. Bland Sutton gave very marked prominence to an ordinance with which I entirely agree, that cases of intra-peritoneal hemorrhage should not be classed as instances of ruptured tubal pregnancy, unless some evidence of fetus or membranes was forthcoming. He was pleased to find so many speakers agreed with him—indeed some speakers seemed to have this string only upon which to play. With all this, as I have said, I entirely agree. But, I want to know what necessity there is for all this energetic denunciation. Where are the cases, and by whom published, which have been regarded and asserted to be cases of ruptured tubal pregnancy, without some evidence of fetus or membranes? I have been watching the literature of this subject most closely for the last fifteen years, and I have not seen any such case published, and it would be very much fairer if the denunciators would point out the delinquents.

Before I go further, let me point out that Mr. Bland Sutton seems to limit his proof to fetus and membranes, but, I suppose, he includes with the latter the placenta; and that it is a mere omission of the pen that he has not specifically mentioned this, the most important proof of all. I point out this markedly because in this reply "he considered it unjustifiable to say in these negative cases that the fetus had been dissolved, but cases in which embryos were not found should be put in a class by themselves until a true cause for them could be found." Here it does really seem as if he had forgotten all about the placenta. Let me say, as the writer who has had now the largest experience of these cases, that in the majority of instances the embryo escapes at the first rupture into the peritoneal cavity and is then lost, is never found and must be digested, for it never does any harm. Similarly the membranes are very rarely seen, but the placenta and its traces are irremovable, because its site is uniformly the bleeding point, and microscopic investigation always reveals its villi permeating the muscular walls of the tube. I have now published forty-five cases of ruptured tubal pregnancy, and in every one this proof has been obtained and can be obtained, for I can put my fingers on every one of the preparations if there is any dispute concerning the fact. Every one of these preparations has been publicly exhibited and discussed at meetings of the British Medical Association, the Midland Medical Society, and the British Gynecological Society. I have, for some time past, ceased to make any communications to the older Metropolitan Societies, for I found that to do so was only to subject myself to much bullying by the chairman ; that to ask for a committee of investigation of my specimens, to corroborate or correct my statements, was only to meet with a court of refusal, and finally my papers were refused publication.

I was specially invited to be present at the hearing of Mr. Bland Sutton's paper, but I need not say that after such treatment I did not avail myself of it. Although I have been for years a member of these societies, I find that the area of publication through them and the advantage of public criticism is denied to me.

To return to the debate of Mr. Sutton's paper : it was most remarkable for the habitual confusion in the minds of most of the speakers, concerning the two varieties of hematocele, intra-peritoneal and extra-peritoneal. I have now seen post-mortem examinations, or surgical operations performed on over eighty cases of extra-peritoneal hematocele (I do not include a few cases where the origin was traumatic, or from a ruptured liver, or from the slipping of the knot on a pedicle,) and in every one of them the cause was a ruptured tubal pregnancy. I may, therefore, be fully excused, when I say that I am entirely sceptical about the statement made by Dr. Cullingworth that intra-peritoneal hematocele might occur from numberless causes. If it does, where are the recorded cases, and what are the proofs?

Dr. Cullingworth speaks of a case in which, on abdominal section, a considerable amount of soft dark clot was found encysted in the peritoneum. He must have meant encysted *by* the peritoneum, that is, the peritoneum raised over it by the effusion—an extra-peritoneal hematocele,—such a case as was immediately after typically described by Dr. Walter, "a dark colored elastic tumor reaching nearly to the umbilicus." In this case, the fetal remains were found at the base, and the case was clearly a broad ligament hematocele, resulting from the rupture of a tubal pregnancy. Dr. Walter's case was precisely the kind in which abdominal section was *not* wanted, quite the case to get well by nature's own process.

The origin of Dr. Walter's case was precisely that of the case jointly alluded to by Dr. Matthews Duncan and Dr. Priestley, in which the patient was suddenly seized with symptoms of hematocele, but where operation was most properly not performed. Eight months after, a dead fetus was extracted from the cavity of the broad ligament, into which the tube had ruptured at the time of the original attack. This is quite the case in which operation at the time of rupture is *not* required, indeed, would be most improper, and the pregnancy ought always be allowed to go on. But the case ought to have been interfered with about six months after, and not eight; for at the end of six months a living child might have been extracted.

Dr. Priestley introduces a new nomenclature for hematocele. He speaks of a "pure" hematocele. What is this? Still more patiently we wait to know when a hematocele may be regarded as impure. He says: "In pure hematocele the patient usually gets well, but if the case were interfered with by operation it was liable to go wrong, either from the direct results of the operation or from subsequent hemorrhage." Now, this is precisely the truth concerning extra-peritoneal or broad ligament hematocele, but why is it to be called "pure" hematocele? "Such cases should be interfered with only when suppuration took place." This may mean when it becomes impure; then we must speak of pure and impure extra-peritoneal hematocele, for intra-peritoneal hematoceles never suppurate.

Of the confusion on this subject indicated by the utterances of Dr. Matthews Duncan, it is almost impossible to speak with patience. He begins his speech with my name, and therefore, I have a right to answer him, and he says: "These enthusiasts for operations taught that there was no such thing as a hematocele, except those produced by ruptured tubal pregnancy, whereas, it was known that hematocele was common even in virgins." By the omission here of the words intra-peritoneal and extra-peritoneal, as defining two wholly different diseases,—different in their origin, in their pathology, and different in what they demand for treatment, wide as the poles asunder,—Dr. Duncan makes these enthusiasts say exactly the opposite of what they have been shouting on the housetops for fifteen years. Dr. Duncan is in the dilemma either of wilful and mischievous representation, or of a dullness of apprehension which is beyond the measurement of mental plummet. I think it must be the latter, for he confesses that he, a man who abhors operations, urged operation in a case of broad ligament hematocele, a proceeding which the enthusiasts condemn. Dr. Duncan is, of course, at liberty to lecture upon this incomprehensible pathology, and to advocate his bad surgery as much as he likes, but he has no right to put them in the mouths of people to object to them, and condemn them unceasingly and emphatically.

Mr. Bland Sutton's communications upon pathology are always interesting and most suggestive, but I have hitherto found them rather of a transcendental than of a practical nature, and his suggestion that the cause of rupture of a tubal pregnancy is an apoplectic change in the ovum belongs rather to this order. In the first place, it cannot possibly be regarded as an explanation; at best, it is only carrying the process one step forward. If the apoplexy is the cause of the rupture, something must be the cause of the apoplexy. It seems to me that the suggestion is clearly based only upon the one case with which he has had experience, and it is diametrically inconsistent with the fact that no preparation has yet been exhibited in which a rupture of a tubal pregnancy has not taken place at a date earlier than the thirteenth week, and the majority of the preparations exhibit no apoplectic conditions whatever. Microscopic investigations of one injected and many non-injected specimens, which I have made, and the results of which I have published over and over again, show that the implantation of the placental villi in the wall of the Fallopian tube involves an enormous increase in the diameter of all the vessels of the structure of the tube, just as results when the placenta implants itself in the uterus, or upon intestine, or upon abdominal wall, or elsewhere. Microscopic investigation, and the complete injection of specimens removed, which I have succeeded in preparing, show that it is at the very spot where the substance of the tubal wall is thinned out by these enlarged vessels that the rupture invariably occurs, and, in the very last specimen which came under my care for the purpose of operation, the rent in the tube is no larger than a pellet of wheat, but the enlarged vessels, at the point of rupture, could be seen, when the specimen was fresh, by a low-power lense with perfect ease, and there was not the slightest apoplectic effusion in the ovum, which is certainly not more than five or six weeks' growth. That a primary effusion of blood into the ovum cavity might precede the actual rupture of the tube now and then, is likely enough, but the suggestion that the cause of rupture is apoplexy of the ovum is simply a proposal to put the cart before the horse.

If Mr. Bland Sutton, and those who took part in the discussion, had read a short paper in the Edinburgh *Medical Journal* for the last month, by Dr. Berry Hart, they could scarcely have failed to have risen to a higher level of accuracy in their statement than was attained. A few sentences from that paper, so absolutely confirmatory of what I have said and repeated at intervals for years past, are worth while quoting here. The first is as follows :

> As all know, the Fallopian tube is, in the vast majority of instances, the starting point of extra-uterine gestation. The most common result of this is that rupture occurs, usually at the second month, through some part of the tube covered by peritoneum, a result almost invariably fatal if left alone, and as invariably curable if operated on in time by abdominal section.
>
> A rarer termination in Fallopian tube gestation is, that further development takes place between the layers of the broad ligament, which become separated to accommodate fetus and placenta. Even here, rupture into the peritoneum may occur ; but if the peritoneum remains intact we may get a continuance of development.

And he goes on to prove the utter inaccuracy of Dr. Matthews

Duncan's statements that the placenta cannot be transplanted, and cannot be moved, and cannot be implanted on cellular tissue, by giving the evidence of his own homalographic sections, which Dr. Duncan quotes, but does not understand. He shows that this displacement may occur, even to the extent of ten inches in distance, and beautifully explains how, "inasmuch as this amount of displacement was accomplished in about as many months, there was never any gross separation, but a slow microscopic progress, which, as we shall see, causes slow blood effusion and organization."

He shows, by drawings of actual specimens, that the large sinuses, which I have described as formed in the muscular walls of the tube, had actually occurred; that the villi, in this way, are compressed, the serotina is destroyed, and, therefore, the relation between mother and fetus so interrupted as to give the explanation of the numerous deaths of extra-uterine children.

This villous destruction and the inevitable epithelial destruction are predisposing in all probability, as is clearly pointed out by Dr. Berry Hart, the causes of the actual apoplectic effusion, which is seen in the future transplantation processes of the placenta, but there is nothing in my own preparations, nor do I see anything in Dr. Berry Hart's observations, which justifies me in the belief that this actual process, to which there exists abundant enough evidence in the post-rupture period of tubal pregnancy, is to be asserted as in any way the immediate or even the indirect cause of tubal rupture. The direct cause is villous implantation, the growth of the sinuses in the muscular coat of the tube, whilst the immediate and direct cause of rupture is generally some slight strain or accident.

7 The Crescent, November 23, 1889.

